# tRNA modification profiles in obligate and moderate thermophilic bacilli

**DOI:** 10.1007/s00792-022-01258-z

**Published:** 2022-02-05

**Authors:** Hovik Panosyan, Franziska R. Traube, Caterina Brandmayr, Mirko Wagner, Thomas Carell

**Affiliations:** 1 Department of Biochemistry, Microbiology and Biotechnology, Yerevan State University, Alex Manoogian 1, 0025 Yerevan, Armenia; 2 Department of Chemistry, Ludwig Maximilian University Munich, Butenandtstrasse 5-13, 81377 Munich, Germany

**Keywords:** tRNA modification, Bacilli, Obligate and moderate thermophiles, Phylogeny marker

## Abstract

Transfer RNAs (tRNAs) are the most ancient RNA molecules in the cell, modification pattern of which is linked to phylogeny. The aim of this study was to determine the tRNA modification profiles of obligate (*Anoxybacillus, Geobacillus, Paragebacillus*) and moderate (*Bacillus, Brevibacillus, Ureibacillus*, *Paenibacillus*) thermophilic aerobic bacilli strains to find out its linkage to phylogenetic variations between species. LC-MS was applied for the quantification of modified nucleosides using both natural and isotopically labeled standards. The presence of m^2^A and m^7^G modifications at high levels was determined in all species. Relatively high level of i^6^A and m^5^C modification was observed for *Paenibacillus* and *Ureibacillus*, respectively. The lowest level of Cm modification was found in *Bacillus.* The modification ms^2^i^6^A and m^1^G were absent in *Brevibacillus* and *Ureibacillus,* respectively, while modifications Am and m^2^_2_G were observed only for *Ureibacillus*. While both obligate and moderate thermophilic species contain Gm, m^1^G and ms^2^i^6^A modifications, large quantities of them (especially Gm and ms^2^i^6^A modification) were detected in obligate thermophilic ones (*Geobacillus, Paragebacillus* and *Anoxybacillus*). The collective set of modified tRNA bases is genus-specific and linked to the phylogeny of bacilli. In addition, the dataset could be applied to distinguish obligate thermophilic bacilli from moderate ones.

## Introduction

In current taxonomy, to reveal evolutionary relationships between prokaryotes several global (evolutionary chronometers) like rRNA molecules, as well as alternative markers (elongation and initiation factors, RNA polymerase subunits, DNA gyrases, heat shock and recA protein) are used (Das et al. 2014). Transfer RNAs (tRNAs) are thought to be among the oldest biological sequences, present at the dawn of life in the last universal common ancestor (Widmann et al. 2010; O’Donoghue et al. 2018; Lei and Burton 2020). tRNAs play an important role in translation, enforcing the genetic code by linking anticodon to amino acid. In addition to their primary role in translation, many bacteria also require aminoacylated-tRNA species to enter the peptidoglycan, antibiotic resistance, antibiotic synthesis and membrane phospholipid modification pathways (Shepherd and Ibba 2015; O’Donoghue et al. 2018). The tRNA genes play also an important role in bacterial conjugation, particularly the integrases recognize the anticodon stem loop region in the tRNA gene for active and site-specific recombination (Shepherd and Ibba 2015).

Due to some reasons (short sequences, involvement in horizontal gene transfer processes, specificity changeable by single point mutation in an anticodon, extensive paralogy through gene duplication) traditionally tRNA sequences are considered as poor candidates for phylogenetic studies (Widmann et al. 2010). However, the question whether more closely related organisms tend to have more similar tRNA modifications remains open.

tRNAs are by far the most extensively modified RNAs. Modifications are post-transcriptionally introduced at precise positions by specific enzymes, and play important roles in folding, stability, identity, in translation fidelity and reading frame maintenance, translational and signaling functions of tRNAs (Shepherd and Ibba 2015). Mature tRNAs are rich in post-transcriptional nucleotide base modifications (O’Donoghue et al. 2018). To date, more than 100 modified nucleosides have been found in tRNA from the three domains of life bacteria, archaea, and eukaryotes (Boccaletto et al. 2018). All post-transcriptional tRNA modifications generate various derivatives of the four common nucleosides adenosine, guanosine, cytidine and uridine, but the exact type of information added by base modification is largely unknown (Globisch et al. 2011; Hori et al. 2018; de Crécy-Lagard et al. 2020).

It was shown also that the collective set of modified tRNA nucleosides is a regulated component of stress response (Chan et al. 2011). Posttranscriptional modification in tRNA is known to play a multiplicity of functional roles, including maintenance of tertiary structure and cellular adaptation to environmental factors such as temperature (Dalluge et al. 1997; Dutta and Chaudhuri 2011; Edwards et al. 2020). The modifications maintaining the conformational flexibility of RNA have been observed in psychrophilic organisms growing under conditions where the dynamics of thermal motion are severely compromised (Dalluge et al. 1997; Lorenz et al. 2017). The role of post-transcriptionally modified nucleosides in the RNA of thermophilic bacteria and archaea in enforcing conformational stability of RNA has been documented (Watanabe et al. 1979; Kowalak et al. 1994).

Recently it was shown some similarities and differences of modified nucleosides in tRNA between moderate thermophiles, thermophiles and mesophiles. Previous studies have shown that the modified nucleosides in tRNA from moderate thermophiles are typically common to those in tRNA from mesophiles. In contrast to mesophilic analogs, the degree of 2’-O-methylation in tRNA from thermophilic *Geobacillus stearothermophilus* is increased at high temperatures (Agris et al. 1973). The studies of tRNA modifications of *Thermus acidophilum* have shown that several modifications (G^+^13 and m^7^G49) stabilize the structure of tRNA and are essential for survival of the organism at high temperatures (Tomikawa et al. 2013). The m^5^s^2^U54 modification has been identified in all *T. thermophiles* (Hori 2019). Although these differences are present, thermophile-specific modified nucleosides (m^5^s^2^U, m^5^Cm, m^1^Im, m^2^_2_Gm, m^2^_7_Gm) have not been found in tRNA from moderate thermophiles (Hori et al. 2018).

Using a parallel systems-type approach Globisch and coauthors (Globisch et al. 2011) shown that the collective set of modified bases is highly species-specific and linked to phylogeny. Authors confirmed also that tRNA modification profiles can be used to differentiate between species, and even pathogenic from non-pathogenic bacteria (Globisch et al. 2011; Koh and Sarin 2018). It was shown also that some tRNA modifications are species-specific (Antoine et al. 2021).

The present study has examined the tRNA modification profiles from a number of bacilli strains at their optimal growth temperatures to clarify of its possibility to differentiate genera of bacilli or distinguish thermophilic and moderate thermophilic bacilli ones. The aim of this study was also to investigate the tRNA modification of different thermophilic bacilli strains to reveal, if the tRNA modification pattern reflects phylogenetic relationships on species level.

To investigate how the set of nucleoside modifications can be used to find out relationships between species the quantified the tRNA modifications by an isotope-dilution-based LC–MS method was used.

## Materials and methods

### Bacterial strains and their growth conditions

The objects of current study were thirteen thermophilic aerobic endospore-forming bacterial strains recently isolated from Armenian geothermal springs and identified based on 16S rRNA gene sequence data as representatives of genera *Anoxybacillus* (*A. flavithermus, A. kamchatkensis*), *Geobacillus* (*G. stearothermophilus, G. thermodenitrificans*), *Paragebacillus* (*P. toebii*), *Bacillus* (*B. licheniformis*, *B. psychrosaccharolyticus*) *Brevibacillus* (*B. thermoruber*) *Ureibacillus* (*U. thermosphaericus*) and *Paenibacillus* (Table 1). All isolates were screened for tolerance to a range of temperatures. Isolates that showed optimal growth at temperatures of 55–65 ºC were defined as obligate thermophiles, whereas those that grew optimally at 50–55 ºC were defined as thermotolerant (Panosyan et al. 2020). The 16S rRNA gene sequences were deposited to Gen-Bank and the accession numbers assigned were as follows: MK418246, MK418249, MK418255, MK418367, MK418381, MK418382, MK418412, MK418552, MK418562, JQ929016, JQ929019-JQ929021.

**Table 1:** List of obligate and moderate thermophilic aerobic bacilli strains studied

Bacilli strains	Temperature range of growth (T_opt_), ^o^C	Accession numbers in GenBank
Obligate thermophilic bacilli		
A.*flavithermus* K-97	45–70 (60)	MK418412
A.*kamchatkensis* J-18	45–70 (65)	MK418562
*Anoxybacillus* sp. H-69	45–70 (65)	MK418382
*G. stearothermophilus* ArzA-3/1	45–75 (65)	MK418367
*G. thermodenitrificans* ArzA-6	45–70 (65)	JQ929020
*Geobacillus* sp. ArzA-7	40–70 (65)	JQ929021
*P. toebii* ArzA-33	45–75 (65)	JQ929016
Moderate thermophilic bacilli		
B.*licheniformis* JG-35	25–60 (55)	MK418552
B.*psychrosaccharolyticus* AkhA-14A	20–55 (50)	MK418255
*Bacillus* sp. B-123a	25–55 (50)	MK418249
*Paenibacillus* sp. ArzA-5	30–55 (50)	JQ929019
*B. thermoruber* H-65	30–60 (50)	MK418381
*U. thermosphaericus* B-119	30–60 (55)	MK418246

All isolates are maintained in the culture collection database of extremophilic microbes at the Department of Biochemistry, Microbiology and Biotechnology of Yerevan State University, Armenia. Glycerol stokes of bacteria were stored at – 80 ºC before usage. During investigation isolates were kept on solid nutrient broth at 4 ºC and were continuously subcultured. Batch cultivation of thermophilic bacilli was carried out using nutrient broth (Difco) under aerobic conditions with shaking at 240 rpm, at pH 7.2 and at optimum growth temperature up to late exponential phase. 1 L nutrient broth was inoculated with 50 mL overnight culture in 5 L wide-mouth Erlenmeyer flasks and incubated at the appropriate conditions until an optical density at λ = 600 nm of 0.8 to 1.0 was attained (late exponential phase of growth). Bacterial culture was transferred to precooled 500 mL centrifugal tubes. The cells were then harvested by centrifugation at 4 ºC temperature, 10,816 g for 10 min, and were immediately suspended in cold buffer 1 containing 0.01 M magnesium acetate, 0.05 M sodium acetate, 0.15 M sodium chloride (pH 4.5).

The suspensions were combined in a 50 mL Falcon tubes and centrifuged at 4 ºC temperature, 3220 g for 30 min. Obtained pellets was kept in at − 80 ºC prior to extraction of tRNA.

### tRNA extraction from bacilli strains

The bacterial pellets were suspended in 15 mL buffer 1 and after addition of 10 mL 80% aq. phenol the suspension was shaken vigorously for 30 min. After centrifugation (4000 g, 30 min) of the obtained mixture, the aquatic layer was collected and treated again with 10 mL 80% aq. phenol. The mixture again was shaken intensively by hands, centrifuged (4000 g, 20 min) and the phenol layers were separated and extracted with 5 mL buffer 1. After vigorous shaking layers were separated by centrifugation (4000 g, 20 min), all aqueous layers were collected and mixed with 10 mL 80% aq. phenol. The mixture was shaken vigorously and centrifuged (4000 g, 20 min) and obtained aqueous layers were treated by 10 mL chloroform. The layers were separated again by shaking and by centrifugation (4000 g, 10 min). After repeating extraction by chloroform, aqueous layers were collected and 20% potassium acetate, pH 4.5 (0.1 vol.) and 12 M lithium chloride were added to a 2 M final lithium chloride concentration.

The mixture was kept on ice for 4 h to precipitate DNA and long chains of RNAs, then centrifuged (20,000 g, 20 min) and absolute ethanol (3 vol.) was added to the supernatant. Mixture was kept at -20 ºC overnight and centrifuged (24,336 g, 60 min) afterwards to obtain crude tRNA. The pellets were dried and kept at -80 ºC until anion exchange chromatography was performed.

### Anion exchange chromatography

All steps on anion exchange chromatography were performed on ice or at 4 ºC. The crude tRNA pellet was dissolved in 10 mL buffer A containing 0.1 M Tris–HCl, pH 7.5, 0.01 M MgCl_2_·6H_2_O. Crude fractions were purified by anion exchange chromatography (DEAE Sepharose fast Flow 5 mL, column volume (CV): 5 mL) utilizing an ÄKTA purifier. Buffer B containing 0.1 M Tris–HCl, pH 7.5, 0.01 M MgCl_2_·6H_2_O, 1 M NaCl was used to create a gradient. The gradient was 5 CV, 0% buffer B; 10 CV, 0% → 40% buffer B; 5 CV, 100% buffer B; 3 CV, 0% buffer B. The fractions eluting at about 20% to 40% buffer B and showing approximately a 2:1 ratio for absorption at λ = 254 nm and λ = 280 nm, respectively, were collected. Absolute ethanol (3 vol.) was added to combined fractions and after keeping at – 20 ºC overnight the mixture was centrifuged (12,000 rpm, 60 min). The obtained pellets were dissolved in ddH_2_O.

### Enzymatic digestion of tRNA

To denature tRNA, the aqueous solution of bulk tRNA (12 µg in 100 µL final volume) was heated to 100 ºC for 30 min and immediately cooled on ice. Afterwards the labeled nucleoside solutions, buffer 2 (300 mM ammonium acetate, 100 mM CaCl_2_, 1 mM ZnSO_4_, pH 5.7) and nuclease S1 (80 units, *Aspergillus oryzae* Sigma Aldrich) were added. After incubation of the mixture for 3 h at 37 ºC the buffer 3 (12 µL, 500 mM Tris–HCl, 1 mM EDTA, pH 8.0), Antarctic phosphatase (10 units), snake venom phosphodiesterase I (0.2 units, *Crotalus adamanteus* venom Sigma Aldrich) were added and incubated for 3 h at 37 ºC to digest completely. DMSO-containing labeled nucleosides were added, followed by centrifugation of the samples (15 min 12,100 g). The supernatant was removed, the volume reduced to 100 μL.

### Liquid chromatography electrospray ionization Tandem mass spectrometry (LC–ESI–MS)

The samples (100 μL injection volume) were analyzed by HPLC–ESI–MS on a Thermo Finnigan LTQ FT-ICR and were eluted by a Surveyor MS pump with a flow of 0.15 mL/min over an Uptisphere120-3HDO column from *Interchim*. The column temperature was maintained at 30 ºC. The buffer C (2 mM ammonium acetate in water, pH 5.5) and buffer D (2 mM ammonium acetate in H_2_O/MeCN 20/80, pH 5.5) were used for eluting.

The gradient was 0 → 55 min; 0% → 8% buffer D; 55 → 100 min; 8% → 60% buffer D; 100 → 102 min; 60% → 100% buffer D; 102 → 120 min; 100% buffer D; 120 → 125 min; 100 → 0% buffer D; 125 → 135 min; 0% buffer D. The elution was monitored at λ = 260 nm. The chromatographic eluent was directly injected into the ion source without prior splitting. Ions were scanned by use of a positive polarity mode over a full-scan range of m/z 200–1000 with a resolution of 30.000. Parameters of the mass spectrometer were tuned with a freshly mixed solution of adenosine (5 mM) in buffer C. The parameters used in this section were sheath gas flow rate, 25 arb; auxiliary gas flow rate, 5 arb; sweep gas flow rate, 5 arb; spray voltage, 5.0 kV; capillary temperature, 200 ºC; capillary voltage, 47 V, tube lens 115 V.

### tRNA modifications used in this study

**Fig. 1:**
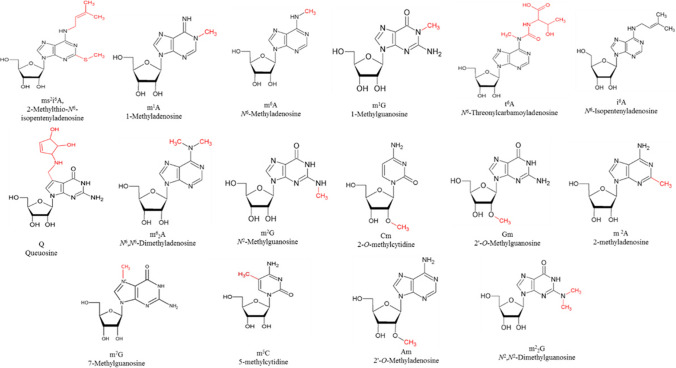
Modified nucleosides synthesized in both natural and isotopically labeled forms for parallel quantification study. The modified nucleosides, ms^2^i^6^A, m^6^A, m^1^G, t^6^A, i^6^A, m^2^A are present at position 37; m^2^G is present at position 9; m^2^_2_G is present at positions 24 and 25; Am present at position 4; Q is present at the wobble position as well as Gm, which is additionally present at position 18; m^1^A is present at positions 14 and 58 (mainly); Cm is present at position 32; m^5^C is present at position 38; m^7^G is present at position 46; m^6^_2_A is present at position 37 (Brandmayr et al. 2012; Duechler et al. 2016)

In total 16 tRNA modifications in both natural and isotopically labeled forms were used in this study (Fig. 1). All modified nucleosides have been synthesized at the Center for Integrated Protein Science, Department of Chemistry, Ludwig Maximilian University Munich, Germany and kindly provided by Prof. T. Carell.

### Calibration curves

Mass calibration curves of the labeled and corresponding unlabeled synthesized nucleosides were obtained at five different concentration ratios. For each concentration an average value of three independent measurements was determined. Each labeled nucleoside solution was mixed with three different concentrations of the corresponding unlabeled nucleosides. The areas of labeled and unlabeled nucleosides from LC-MS measurements were determined using the *Qualbrowser* (Xcalibur) program by extraction of the accurate mass range with a mass filter out of the total ion count. The linear fits of the determined area ratios with the amount ratios gave R^2^-values of minimum 0.9992. The linear fit equations were used for calculation of the exact nucleoside contents in bulk tRNA samples. Synthetic labeled nucleosides were added to the digest solutions and the areas of labeled and unlabeled nucleosides were determined as described above. The amount of each nucleoside was calculated from the obtained area ratios and the linear fit equations of the calibration curves. The level of tRNA modification has been quantified by ratio of mols (modification) / mols (A nucleoside) per 1000 tRNAs.

### Phylogenetic analysis based on 16S rRNA genes

Total bacterial genomic DNA was extracted, PCR using by universal primer pairs 27f (5'-GAGTTTGATCCTGGCTCA-3') and 1525r (5'-GAAAGGAGGAGATCCAGCC-3') (*Escherichia coli* numbering) to amplify of 16S rRNA genes and sequencing of bacterial 16S rDNA amplicons were performed as described by Panosyan et al. (2020). Alignment of sequences for phylogenetic analysis was made using ClustalW (Thompson et al. 1994). A phylogenetic tree of the strains was constructed using the neighbor-joining method (Saitou and Nei 1987) with the MEGA X software (Kumar et al. 2018). The evolutionary distances were computed using the Maximum Composite Likelihood method (Tamura et al. 2004) and are in the units of the number of base substitutions per site. Confidence in branching points was determined by bootstrap analysis (1000 replicates) (Felsenstein 1985).

### Species clustering based on quantitative tRNA modification data

Hierarchical clustering was performed in Perseus (version 1.6.14.0) (Tyanova et al. 2016) on the quantified RNA modifications. First, the RNA modifications were normalized using the Z-Score function (rows = bacterial strain, column = RNA modification; matrix access column). Then hierarchical clustering (columns and rows) was performed using Euclidian distance, average linkage, no constraints with maximal 10 iterations and 300 clusters as set parameters.

## Results

### tRNA modification pattern of studied thermophilic bacilli

We analyzed the post-transcriptional tRNA modification pattern of 13 bacilli species belonged to genera *Anoxybacillus*, *Geobacillus, Paragebacillus, Bacillus*, *Brevibacillus Ureibacillus* and *Paenibacillus.* Bulk tRNA was extracted from bacilli grown in complex media under optimum conditions. This was followed by digestion and subsequent analysis of the resulting nucleoside mixture using quantitative LC–MS. The results for all investigated species are presented in Table 2.

**Table 2:** tRNA modification pattern of all the samples investigated

Species of bacilli			tRNA modifications level*													
	m^1^A	m^6^A	m^6^_2_A	m^2^A	t^6^A	i^6^A	ms^2^i^6^A	m^1^G	m^2^G	m^7^G	m^2^_2_G	Q	m^5^C	Am	Cm	Gm
*A. flavithermus* K-97	15.67	1.23	1.20	281.08	5.66	0.23	7.50	13.60	5.52	28.90	0.00	13.30	1.55	0.00	1.58	26.27
*A. kamchatkensis* J-18	19.34	1.39	0.91	215.72	6.39	0.37	12.07	13.94	6.82	29.46	0.00	19.76	0.94	0.00	1.53	26.74
*Anoxybacillus* sp. H-69	20.79	1.49	0.46	293.94	6.21	0.20	11.69	16.48	7.50	32.74	0.00	21.74	0.56	0.00	1.54	29.99
*G. stearothermophilus* ArzA-3/1	16.07	1.97	0.97	247.34	12.88	0.30	8.25	16.41	4.72	46.41	0.00	17.82	0.71	0.00	1.45	55.13
*G. thermodenitrificans* Arz-6	21.34	1.29	0.56	342.97	12.20	1.04	11.59	16.88	4.53	33.93	0.00	5.55	0.94	0.00	2.00	38.55
*Geobacillus* sp. Arz-7	22.78	1.33	0.71	406.71	8.10	1.00	12.26	17.39	27.80	31.97	0.00	14.90	1.07	0.00	2.47	39.48
*P. toebii* Arz-33	10.79	0.84	2.01	231.17	6.86	0.09	5.70	8.30	3.88	21.93	0.00	6.76	2.06	0.00	2.22	31.37
*B. licheniformis* JG-35	18.83	1.46	0.15	231.83	6.56	3.85	4.72	12.60	6.82	40.50	0.00	17.90	0.29	0.00	0.87	4.43
*B. psychrosaccharolyticus* AkhA-14A	18.23	1.48	0.26	296.35	11.35	0.53	8.39	13.62	5.94	37.28	0.00	17.31	0.00	0.00	0.69	6.31
*Bacillus* sp. B-123a	7.56	0.49	1.37	162.80	6.64	0.50	3.25	4.72	2.70	13.66	0.00	3.91	2.43	0.00	2.35	3.29
*Paenibacillus* sp. ArzA-5	19.03	1.18	0.79	383.03	8.51	5.96	3.03	6.13	18.16	25.18	0.00	12.57	0.64	0.00	1.83	2.36
*B. thermoruber* H-65	2.66	0.24	3.18	78.25	4.55	0.22	0.76	0.00	5.68	10.43	0.00	2.78	2.21	0.00	1.15	6.48
*U. thermosphaericus* B-119	5.00	0.48	2.53	37.59	5.08	2.31	0.00	3.62	3.61	10.75	1.47	4.49	7.59	3.62	5.19	8.34

^*^The level of tRNA modification is presented as ratio of mols(modification)/ mols(A nucleoside) per 1000 tRNAs

The first key result from this data is that the modifications vary dramatically between species. For example, i^6^A varies from 0.09 in the *Geobacillus thermodenitrificans* Arz-6 to 5.96 in the *Paenibacillus xylanilyticus* Arz-3, and t^6^A varies from 4.55 in the *B. thermoruber* H-65 to 12.88 in *G. stearothermophilus* ArzA-3/1. m^2^A and m^7^G modifications were presented at high levels in all the species studied. High amounts of certain modifications characterize particular groups of bacilli. Thus, relatively high amount of m^1^G modification were found for representatives of *Geobacillus*, while Q was generally high in *Anoxybacillus.* Gm was generally high in representatives of genera *Geobacillus*, *Parageobacillus* and *Anoxybacillus*. Particularly, representatives of the genus *Geobacillus* were characterized by highest level of Gm modifications. Relatively high level of i^6^A and m^5^C modifications were observed for *Paenibacillus* and *Ureibacillus*, respectively. m^5^C was absent only in *B. psychrosaccharolyticus* AkhA-14A. Relatively high amount of m^2^G was found for *Geobacillus* sp. Arz-7 and *Paenibacillus* sp. ArzA-5.

High amount of m^1^A modification was observed for representatives of *Geobacillus*, *Anoxybacillus*, *Paenibacillus* and *Bacillus,* while it was lower in *Ureibacillus* and *Brevibacillus.* Cm modification was highest in *U. thermosphaericus* B-119. Large quantities of ms^2^i^6^A modification were also detected in the representatives of genera mainly belonging to *Geobacillus* and *Anoxybacillus.*

According obtained data the m^6^A, i^6^A, m^6^_2_A, m^5^C and Cm modifications are present at low quantities (≤ 7.59) in all the species studied. Low amounts of some modifications were typical for particular groups of bacilli. Thus i^6^A modification was presented at low levels in representatives of *Anoxybacillus, Parageobacillus, Brevibacillus,* as well as of *G. thermodenitrificans* Arz-6 and *Bacillus* sp. B-123a. For *Ureibacillus* lowest level was observed in case of m^6^A modification, while the lowest level of remained species belonged to genera *Geobacillus* and *Bacillus* was observed for m^6^_2_A modification.

Lowest level of m^5^C modification was determined only for *Paenibacillus.* Gm was generally low in representatives of genera *Bacillus, Paenibacillus, Ureibacillus* and *Brevibacillus.* Our data show that m^6^A, m^1^G, t^6^A, ms^2^i^6^A, m^2^A and m^7^G modifications were present at low levels in *Ureibacillus* and *Brevibacillus* species. The lowest level of Q modification was observed for *Brevibacillus*. Moreover, m^1^G and ms^2^i^6^A modifications were present in all bacteria except *Brevibacillus* and *Ureibacillus*, respectively. In contrast to this, m^6^_2_A and m^5^C modifications were more distributed among *Ureibacillus* and *Brevibacillus*.

Among N^6^-isopentenyladenosine derivatives (i^6^A and ms^2^i^6^A), i^6^A itself is present in all the species investigated. The ms^2^i^6^A modification was absent only in *Ureibacillus sp.* B-119. On the other hand, only in *U. thermosphaericus* B-119 the modifications Am and m^2^_2_G were exclusively observed. Additionally, m^1^G is present in all bacteria, except in *Brevibacillus* sp. H-65.

Based on absolute calculation high quantities of m^1^A, m^6^A, m^1^G, t^6^A, i^6^A, ms^2^i^6^A, Q, m^2^G, Gm, m^2^A and m^7^G modifications were more typical for obligate thermophiles. Highest values of m^1^A, m^6^A, m^1^G, t^6^A, ms^2^i^6^A, m^2^G, Gm, m^2^A and m^7^G were observed for *Geobacillus*, while Q was highest for *Anoxybacillus.* In contrast to this, lowest quantities of m^1^A, m^6^A, m^1^G, t^6^A, ms^2^i^6^A, Q, m^6^_2_A, m^2^G, Cm, Gm, m^2^A m^7^G and m^5^C modifications were observed for moderate (*Bacillus*, *Brevibacillus*, *Ureibacillus* and *Paenibacillus*) bacilli. Lowest amount of i^6^A modification was found only for *Parageobacillus.* Modifications Cm, m^5^C, Am and m^2^_2_G with highest and m^1^A, m^1^G, t^6^A and m^2^A with lowest quantities and absence of ms^2^i^6^A were observed for *Ureibacillus.* Highest level of i^6^A and m^6^_2_A, modifications were observed for *Paenibacillus* and *Brevibacillus,* respectively. m^6^A, ms^2^i^6^A, Q and m^7^G modifications were presented at lowest level in *Brevibacillus,* while Gm was the lowest modification obtained for *Paenibacillus.*

### Species clustering based on 16S rRNA gene sequences and quantitative tRNA modification data

**Fig. 2:**
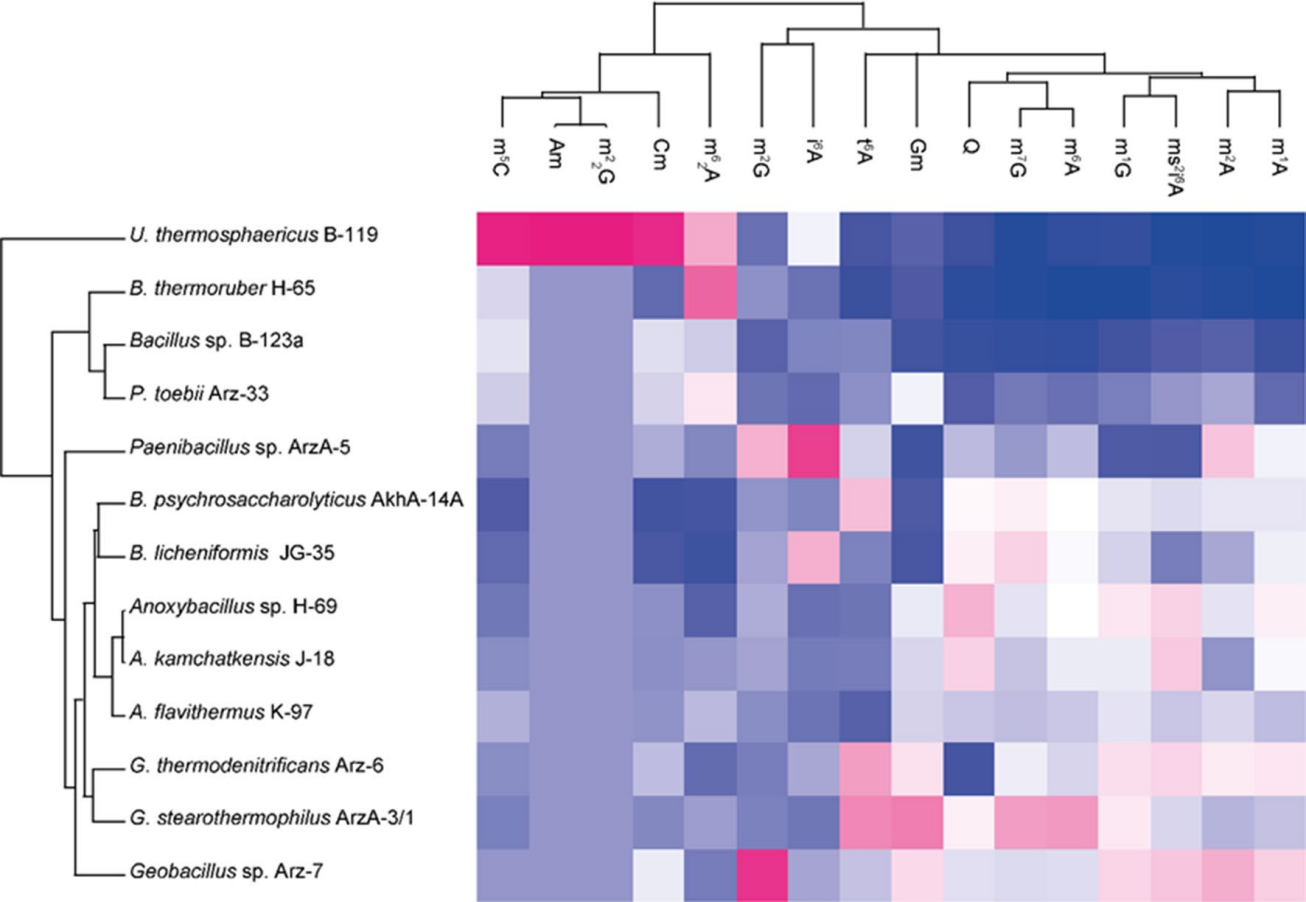
Hierarchical clustering of species and tRNA modifications. The Heat map was calculated using Euclidian distance for species and tRNA modifications based on Z-score normalized tRNA modification levels. Z-score ranges from − 1.91 (dark blue) to 3.33 (bright magenta)

In this study a hierarchical clustering algorithm to the data was applied to statistically analyze the differences in modification levels between species (Fig. 2). According to the obtained results bacilli genera are clearly separated from each other and even species from the same genus can clearly be differentiated.

**Fig. 3:**
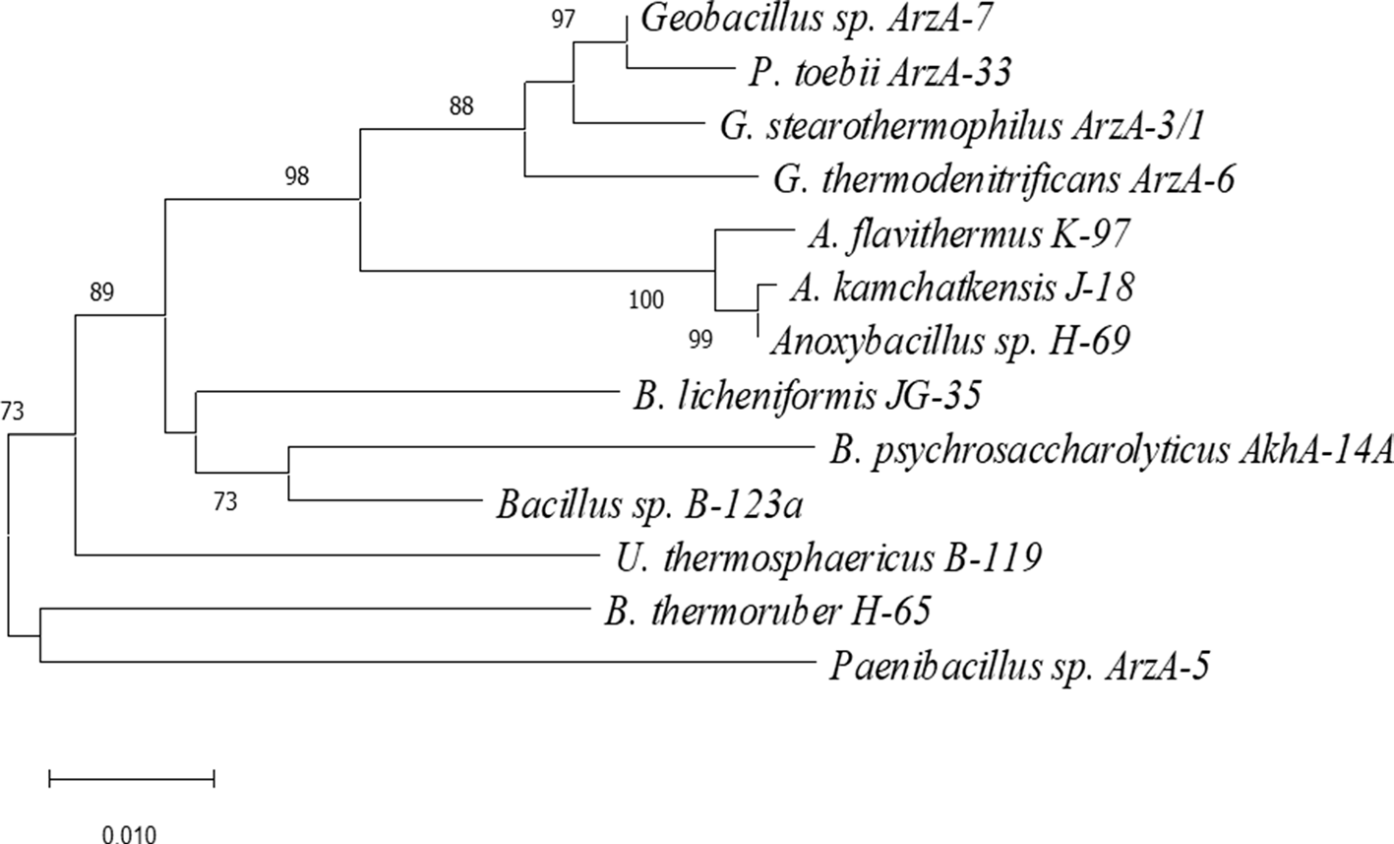
Phylogenetic tree based on nearly complete 16S rRNA gene sequences, showing the relationships between isolated of *Anoxybacillus Geobacillus, Paragebacillus, Bacillus*, *Brevibacillus*, *Ureibacillus* and *Paenibacillus.* Evolutionary analyses were conducted in MEGAX using the neighbor-joining method. The percentage of replicate trees (> 73%) in which the associated taxa clustered together in the bootstrap test (1000 replicates) is shown next to the branches. Scale bar represents 0.01 substitutions per site

To compare obtained data from clustering of tRNA modifications, the phylogenetic tree based on orthologous 16S rRNA genes for all species present in our study was constructed (Fig. 3). The phylogenetic tree confirmed that representatives of genera *Geobacillus* and *Parageobacillus* are part of the same cluster, indicating their close relationship. Each genus composes a separate cluster and is clearly distinct from others. Obviously, clusters including obligate thermophilic species (*Geobacillus*, *Parageobacillus* and *Anoxybacillus* are distinctly separated from clusters with moderate thermophilic species.

## Discussion

It was shown earlier that some modifications (m^1^G and t^6^A) are present in organisms of all three domains of life and probably have evolved early (Grosjean et al. 1995; Globisch et al. 2011). Therefore, they had to develop early during the evolution of life. Moreover, t^6^A seems to be more abundant in Gram-positive than in Gram-negative bacteria (Globisch et al. 2011). Our data show that the m^2^A and m^7^G modifications are present at high levels in all bacilli species studied. Other modifications like m^1^A, Q and Gm are also presented at relatively high level. These modified nucleosides along with t^6^A and some others (m^6^A, i^6^A, m^6^_2_A, m^2^G, Cm), are the tRNA nucleosides present in all seven genera studied. m^1^G modification was missed only for *B. thermoruber* H-65. We conclude the mentioned modifications with high level presence in all the species studied belong to the oldest RNA modifications which probably contributed to the very early development of endospore-forming bacteria. These initial observations show evolutionary relationships related not only to the presence, but also to the abundance of each tRNA modification.

In contrast to *Geobacillus*, and *Anoxybacillus* level of m^1^A and m^1^G modifications for *P. toebii* Arz-33 is lower. The lowest level of i^6^A modification was registered again for *P. toebii* Arz-33, while m^6^_2_A modification is comparably higher in it.

*Paenibacillus* sp. ArzA-5 by highest content of i^6^A and by lowest content of Gm modifications easily can be differentiated from all studies species. Only the *Bacillus* species were not significantly distinguishable by their modifications pattern.

The presented results offer a deeper insight into the evolution of tRNA modifications, and shows that they characterize genus/species at a very fine level and are linked to phylogenetic variation of endospore-forming bacteria.

Inspired from these differences between genera/species we applied a hierarchical clustering algorithm which helped to group together species with similar modification patterns (Fig. 2). Results were compared with data of clustering of species based on orthologous 16S rRNA genes (Fig. 3). We investigated the tRNA modification levels of different bacilli strains in a comparative analysis to reveal, if the tRNA modification patterns are conserved, random, or if the collection mirrors phylogenetic relationships. Surprisingly, the two independent clustering experiments provide almost similar grouping patterns, thus confirming that modified nucleoside levels are closely linked to genetic variation of species. *U. thermosphaericus* B-119 was clearly separated from other bacilli, and there was also a clear distinction between obligate (*Anoxybacillus Geobacillus, Paragebacillus*) and moderate (*Bacillus*, *Brevibacillus*, *Ureibacillus* and *Paenibacillus*) thermophilic bacilli (Fig. 3.). Similarly, the bacilli cluster in correlation with the phylogenetic tree, with a distinction between bacilli species, and even a separation between obligate and moderate thermophilic ones. The obligate thermophilic bacilli clustering also shows similarity to the phylogenetic groupings, with the exception of *P. toebii* Arz-33, which clusters with moderate thermophiles rather than the more closely related *Geobacillus* and *Anoxybacillus*, thus highlighting its ambiguous character. The closely related genera *Bacillus, Brevibacillus* and *Paenibacillus* are distinguishable too.

Based on obtained results obligate and moderate thermophilic bacilli are clearly distinguishable. While both obligate and moderate thermophilic species contain Gm, m^1^G and ms^2^i^6^A modifications, large quantities of them (especially Gm and ms^2^i^6^A modification) were detected in obligate thermophilic ones (representatives of genera *Geobacillus, Paragebacillus* and *Anoxybacillus*) optimum temperature of which is ≥ 60 °C*.* Moreover, among obligate thermophilic bacilli, representatives of the genus *Geobacillus* are characterized by highest Gm modification (1.6 times more). High level of i^6^A modification was found for moderate thermophiles. However, in general this analysis provides a novel possibility for differentiation between obligate and moderate thermophilic bacilli. Earlier it was shown existence of some differences between thermophiles and mesophiles. Thus, Agris and coauthors (Agris et el. 1973) shown that degree of 2’-O-methylation in tRNA from *G. stearothermophilus* is increased at high temperatures which is in agreement with the data obtained. The tRNA stabilization strategies by modified nucleosides based on thermophile-specific modification such as m^5^s^2^U54 and on 20-O-methylations at multiple positions in tRNA are known among extreme-thermophiles (*Thermus thermophiles*) and hyper-thermophiles (*Pyrococcus horikoshii*) (Hori et al. 2018).

In summary, we have measured 16 tRNA modifications in 13 species quantitatively. The presented results offer a deeper insight into the evolution of tRNA modifications, and shows that they characterize species at a very fine level and are linked to phylogenetic variation of endospore-forming aerobic bacteria. Additionally, the data can be used to differentiate between genera of aerobic bacilli, and even to distinguish obligate and moderate thermophilic bacilli species. This work reports, for the first time, a complex study of tRNA modification pattern of bacilli and possibility to distinguish bacilli genera/species based on modification levels.
